# Maternal body fluid lncRNAs serve as biomarkers to diagnose ventricular septal defect: from amniotic fluid to plasma

**DOI:** 10.3389/fgene.2023.1254829

**Published:** 2023-09-06

**Authors:** Huaming Wang, Xi Lin, Xinda Wang, Xinxiu Liu, Shaozheng He, Guorong Lyu

**Affiliations:** ^1^ Department of Ultrasound, The Second Affiliated Hospital of Fujian Medical University, Quanzhou, Fujian, China; ^2^ Department of Diagnostic Radiology, Fujian Cancer Hospital of Fujian Medical University, Fuzhou, Fujian, China; ^3^ Department of Radiology, Quanzhou First Hospital Affiliated to Fujian Medical University, Quanzhou, Fujian, China; ^4^ Department of Medical Ultrasound, The First Affiliated Hospital of Fujian Medical University, Fuzhou, Fujian, China

**Keywords:** long non-coding RNA, ventricular septal defect, maternal body fluids, prenatal diagnosis, epigenetic

## Abstract

**Background:** Maternal body fluids contain abundant cell-free fetal RNAs which have the potential to serve as indicators of fetal development and pathophysiological conditions. In this context, this study aimed to explore the potential diagnostic value of maternal circulating long non-coding RNAs (lncRNAs) in ventricular septal defect (VSD).

**Methods:** The potential of lncRNAs as non-invasive prenatal biomarkers for VSD was evaluated using quantitative polymerase chain reaction (qPCR) and receiver operating characteristic (ROC) curve analysis. The biological processes and regulatory network of these lncRNAs were elucidated through bioinformatics analysis.

**Results:** Three lncRNAs (LINC00598, LINC01551, and GATA3-AS1) were found to be consistent in both maternal plasma and amniotic fluid. These lncRNAs exhibited strong diagnostic performance for VSD, with AUC values of 0.852, 0.957, and 0.864, respectively. The bioinformatics analysis revealed the involvement of these lncRNAs in heart morphogenesis, actin cytoskeleton organization, cell cycle regulation, and protein binding through a competitive endogenous RNA (ceRNA) network at the post-transcriptional level.

**Conclusion:** The cell-free lncRNAs present in the amniotic fluid have the potential to be released into the maternal circulation, making them promising candidates for investigating epigenetic regulation in VSD.

## 1 Introduction

The development of the fetal heart is a complex and dynamic process; even a slight perturbation can be catastrophic and lead to a range of congenital heart defects (CHDs). Ventricular septal defect, one of the most common forms of CHD, has an occurrence rate of 4.3 in 1,000 births (Liu et al., 2019). With surgical intervention, infants with VSD have satisfactory long-term survival. However, they are still confronted with the risk of various short- and long-term complications (Ren et al., 2021). Therefore, novel insights into the underlying triggers of VSD are urgently needed to develop better diagnostic and therapeutic strategies for VSD. According to epidemiological investigations, abnormal heart development is associated with the accumulation of numerous molecular alterations (Deita et al., 2021). Although aberrant genetic alterations, such as aneuploidy, mutations, insertions, and deletions, are considered to be closely related to CHDs, they cannot be accounted for entirely by genetic inheritance (Gutierrez et al., 2022). In recent years, numerous studies have shown that epigenetic modifications play a significant role in regulating gene expression, which are also critical mediators for driving and maintaining heart development (Hou et al., 2020).

There are three main types of epigenetic modifications: non-coding RNAs (ncRNAs), histone modifications, and DNA methylation. Kan et al. (2022) revealed that long non-coding RNAs function as epigenetic regulators implicated in fetal heart gene expression and pathological processes of VSD. Several studies have found that differential expression of lncRNAs in CHD patients will participate in heart development by affecting cardiomyocyte proliferation. For instance, lncRNA SAP30-2:1 is downregulated in CHDs and regulates cell proliferation by targeting HAND2 (Ma et al., 2021); lncRNA-HA117 is upregulated in the myocardial tissue of Tetralogy of Fallot (ToF) children and negatively correlated with the prognosis (Wang et al., 2018). At the pre-transcriptional level, lncRNA can act as an enhancer or alter DNA methylation to inhibit cardiomyocyte differentiation and maintain cardiac stem cell pluripotency (Devaux et al., 2015; Zhang et al., 2019). At the post-transcriptional level, lncRNA-associated competitive endogenous RNA (ceRNA) is an essential epigenetic patterning of the genome. In ceRNA patterning, lncRNA binds to miRNAs to regulate the expression of downstream messenger RNA, thus causing CHDs to occur. For example, lncRNA FGD5-AS1 plays a repressive role in heart development through binding with has-miR-421 to regulate the expression of SMAD4 (Zhang et al., 2021). Given the adverse effects of VSD and the crucial role of lncRNAs in cardiac development, comprehending the regulatory mechanisms of these lncRNAs is central to the diagnosis and treatment of VSD.

Maternal body fluids are abundant in cell-free fetal DNAs and RNAs which can reflect fetal development and pathophysiological status (Wagner et al., 2019). Biomarkers from amniotic fluid and maternal plasma are considered powerful diagnostic tools for assessing fetal health and disease. Studies have shown that amniotic fluid ncRNA profiles play a significant role in clinical prediction in fetal infection and twin–twin transfusion syndrome recipients (Willner et al., 2021; Yoshikawa et al., 2021). In our previous publication, we found that some amniotic fluid lncRNAs (LINC00598, LINC01551, PWRN1, and GATA3-AS1) were differently expressed in VSD fetuses, which may be potential biomarkers for VSD fetuses (Wang et al., 2023). However, it remains to be investigated whether the expression of lncRNAs associated with VSD is consistent in maternal plasma and amniotic fluid. In addition, amniotic fluid biomarkers are not clinically appropriate as routine methods due to the complications of amniocentesis. Recently, research has shown that maternal circulating microRNAs can be used as non-invasive biomarkers for the prenatal diagnosis of CHDs (Gu et al., 2019). This motivates the possibility that circulating lncRNAs have the same potential.

Therefore, in the present study, we used qPCR to verify the expression of LINC00598, LINC01551, PWRN1, and GATA3-AS1 in maternal plasma. Subsequently, the integrated bioinformatics methods, including coding–non-coding gene co-expression network (CNC), weighted gene co-expression network analysis (WGCNA), and ceRNA regulation network, were used to predict the hypothetical functions and mechanisms of these lncRNAs in VSD.

## 2 Materials and methods

### 2.1 Patient sample collection

All pregnant women were recruited from January 2021 to February 2023 at The Second Affiliated Hospital of Fujian Medical University. All pregnant women were assessed for prenatal ultrasound and echocardiography. Pregnant women who carried a VSD fetus were categorized into the VSD group, while those who carried a normal fetus were grouped into the control group. Exclusion standards included multiple pregnancies, pregnancies with placental anomalies, and fetuses with extracardiac anomalies. All fetuses were confirmed by postpartum ultrasound. Both groups of pregnant women did not report CHD risk factors, such as alcoholism, smoking, diabetes, hypertension, and pharmaceutical history. All pregnant women did not suffer from pregnancy complications, such as gestational diabetes mellitus and preeclampsia. This study was approved by the Ethics Committee of The Second Affiliated Hospital of Fujian Medical University (No. 2021-73) and conducted in accordance with the principles of the World Medical Association Declaration of Helsinki.

Maternal blood samples were collected from all pregnant women on an empty stomach. The fasting venous blood (5 mL) was collected from individuals with EDTA tubes. Blood samples were centrifuged at 3,000 g for 10 min. The isolated plasma was centrifuged at 13,500 g for 10 min to remove cell fragments, and then the supernatant samples were stored at −80°C until RNA extraction.

### 2.2 RNA extraction and quantitative polymerase chain reaction

According to the manufacturer’s protocol, total RNA was extracted from plasma supernatant using TRIzol LS reagent (Invitrogen, Carlsbad, CA). Subsequently, NanoDrop ND-1000 was used to measure the RNA quantities and qualities. qPCR was performed according to PrimeScript™ RT Reagent Kit with gDNA Eraser (Takara, Dalian, China) and TB Green^®^ Premix Ex Taq™ II (Takara, Dalian, China). Briefly, 1°μg of total RNA was used to synthesize cDNA. Then, the cDNA was amplified and quantified under the following cycling parameters: 95°C (30°s) at the first step; 95°C (5°s) and 60°C (34°s) for 40 cycles at the second step; and 95°C (15°s), 60°C (60°s), and 95°C (15°s) at the third step. The 2^−ΔΔCT^ method was performed to calculate the gene expression levels. The sequences of the primers used are shown in Supplementary Table S1. The relative expression levels of lncRNAs were normalized to the expression levels of GAPDH and measured in triplicate for each sample.

### 2.3 Construction of the CNC network

It is known that lncRNAs can function by regulating mRNA expression. To further reveal the function of the aforementioned lncRNAs, co-expression analysis was used to unearth mRNAs associated with these lncRNAs. First, the mRNA expression data were obtained from our previous research (GEO: GSE204935), which contained 11 VSD fetuses and 11 normal fetuses (Wang et al., 2023). Second, based on the previously described co-expression analysis of lncRNAs and mRNAs, we constructed a CNC network by calculating Pearson’s correlation coefficient. Genes with Pearson’s correlation coefficients above 0.90 and *p*-values below 0.05 were selected for the network. These results were visualized using the Cytoscape (v2.8.2) program.

### 2.4 Functional enrichment analysis

In the cell, active transcription and translation of mRNAs directly control the expression of localized proteins and subsequent signal regulation. To identify the potential role of lncRNAs, Gene Ontology (GO) and Kyoto Encyclopedia of Genes and Genomes (KEGG) analyses were performed to reveal the key functional classifications, relationship networks, and disease information for mRNAs in the CNC network. The GO and KEGG categories were classified by Fisher’s exact test. The threshold of significance was defined by the false discovery rate (FDR) and *p*-value.

### 2.5 Weighted correlation network analysis and identification of clinically related key modules

WGCNA, a systems biology approach, can be used to identify genetic modules of highly correlated genes and related modules to external sample traits. In order to reveal the VSD-related eigengene network, the WGCNA package of R was used to construct gene association patterns between different samples according to mRNA expression data (Langfelder et al., 2008). First, the top 75% of genes were screened according to median absolute deviation (MAD). Second, the weighted co-expression relationship among all genes was evaluated by calculating Pearson’s coefficient. Third, to ensure the construction of a scale-free network, we found a soft-threshold power β using the “pickSoftThreshold” function. Fourth, a topological overlap matrix separates all genes into multiple gene modules to represent network connectivity. The genes contained in each gene module are well correlated. The dynamic branch cut method was used to conduct hierarchical clustering dendrograms, and then each module was represented by a designated color (Langfelder and Horvath, 2008). Finally, key modules significantly associated with clinical traits were further identified by calculating module–trait relationships and gene significance. To further identify candidate genes of VSD, genes whose gene significance (GS) > 0.3 and module membership (MM) > 0.8 were defined as interesting genes in the key module.

### 2.6 Construction of the lncRNA–miRNA–mRNA interaction network

One of the potential mechanisms of lncRNAs is working as miRNA sponges to affect mRNA’s function at the post-transcriptional level. Therefore, we constructed a ceRNA network to explore the potential role of lncRNAs in VSD. First, the positive lncRNA–mRNA pairs were identified based on the CNC network. Second, the miRcode and TargetScan databases were used to predict miRNA targets on lncRNAs and mRNAs, respectively. Finally, the intersection of lncRNA-predicted miRNAs and mRNA-predicted miRNAs was used to construct the lncRNA–miRNA–mRNA interaction network. The Cytoscape program was used to visualize the ceRNA network.

### 2.7 Statistical analysis

All data were analyzed using R software (4.1.3), SPSS (21.0), Stata (15.1), and GraphPad Prism (7.0). The quantitative data are expressed as the average ± standard deviation. The statistical analysis of age, gestational weeks, and gene expression levels was performed using the Student’s t-test. The receiver operating characteristic curve was constructed to evaluate the diagnostic value of lncRNAs. The significance of GO or KEGG enrichment was evaluated by Fisher’s exact test. *p* < 0.05 was considered to indicate a statistically significant difference.

## 3 Results

### 3.1 Clinical characteristics

In total, 22 pregnant women with VSD fetuses and 16 pregnant women with normal fetuses met the criteria and were included in the study. There were no differences in maternal age, the number of pregnancies, gestational age, and body mass index (BMI) between the two groups (Table 1; Supplementary Table S2). Maternal blood samples were collected from all pregnant women on an empty stomach.

**TABLE 1:** Clinical characteristics of the VSD and control groups.

Characteristic	VSD group (*n* = 22)		Control group (*n* = 16)	*p*-value
Maternal age (years)	29.95 ± 3.0		30.75 ± 4.5	0.52
Gestational age (weeks)	21.14 ± 0.9		20.69 ± 0.8	0.12
Number of deliveries	1.86 ± 0.8		2.13 ± 0.7	0.29
Body mass index (kg/m^2^)	22.00 ± 1.1		21.88 ± 1.1	0.74

### 3.2 Verification of lncRNAs in maternal plasma

**FIGURE 1:**
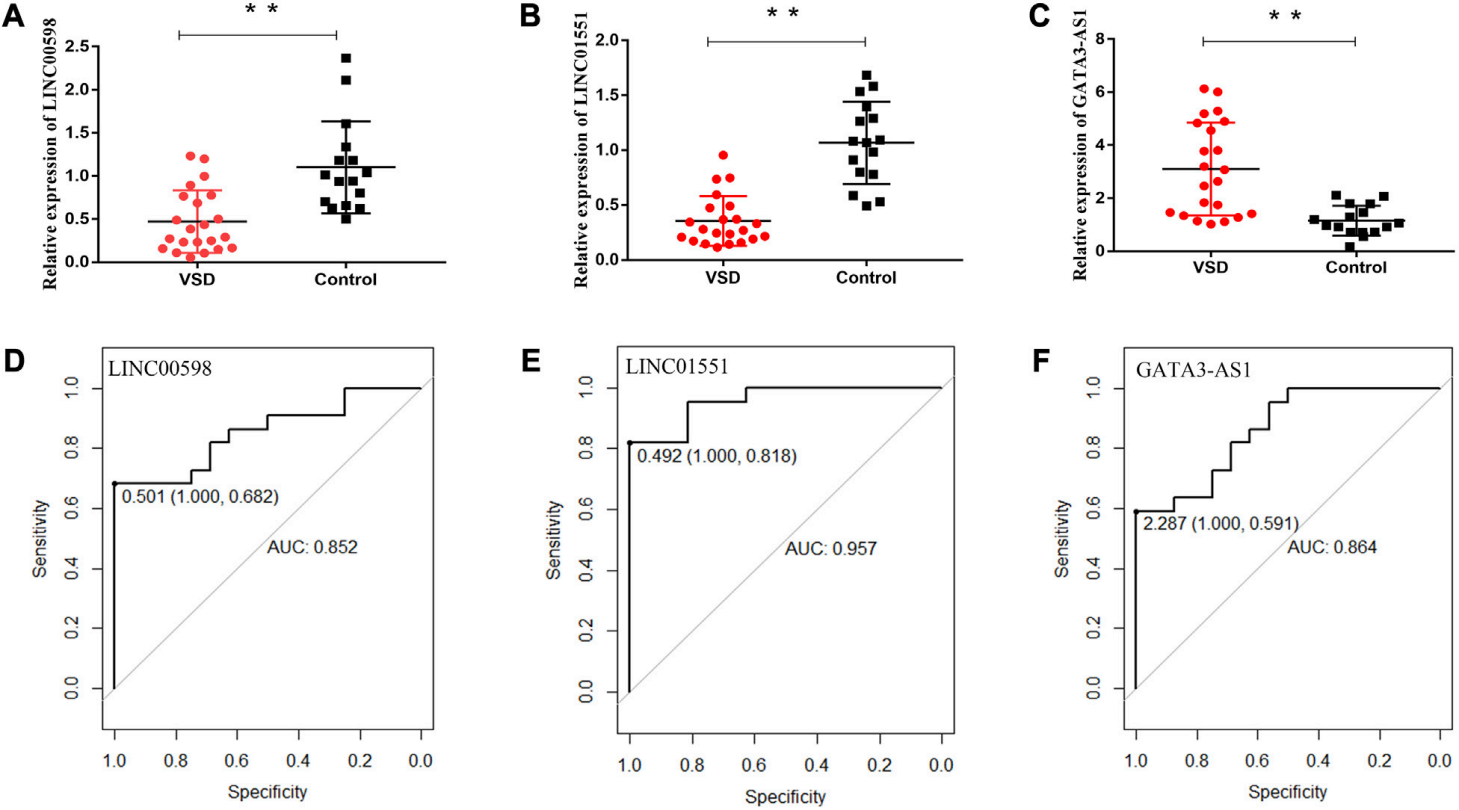
Potential of relevant RNAs as non-invasive prenatal biomarkers for VSD. Relative expression levels of lncRNAs in maternal plasma and the receiver operating characteristic (ROC) curves of LINC00598 **(A, D)**, LINC01551 **(B, E)**, and GATA3-AS1 **(C, F)**. * indicates *p* < 0.05, and ** indicates *p* < 0.01. AUC, area under the ROC curve.

To explore whether the expression of VSD-related genes is consistent in maternal plasma and amniotic fluid, four lncRNAs (PWRN1, GATA3-AS1, LINC00598, and LINC01551) were selected for qPCR. The qPCR results showed that three lncRNAs were consistent with amniotic fluid, including LINC00598, LINC01551, and GATA3-AS1. Subsequently, the ROC curve was drawn to evaluate the potential of these lncRNAs as biomarkers for VSD. The result showed that these lncRNAs have a good diagnostic performance on VSD with AUC values of 0.852, 0.957, and 0.864, respectively (Figure 1; Table 2). Furthermore, we assign an equal weight to each of the multiple lncRNAs to construct a combined model. The results show that the combined model with three lncRNAs has an AUC of 0.994, which is better than the combined model with only two lncRNAs. (Supplementary Figure S1). The RNA quantification and quality assurance are shown in Supplementary Table S3. In addition, the raw data of qPCR are shown in Supplementary Tables S4–S6.

**TABLE 2:** Differential expression of lncRNAs in maternal plasma.

lncRNA	VSD group (*n* = 22)	Control group (*n* = 16)	Fold change	*p*-value
	Mean SD	Mean SD		
LINC00598	0.47 0.36	1.11 0.53	0.42	0.000
GATA3-AS1	3.10 1.75	1.16 0.56	2.67	0.000
LINC01551	0.35 0.23	1.07 0.37	0.33	0.000

### 3.3 Construction of the CNC network

**FIGURE 2:**
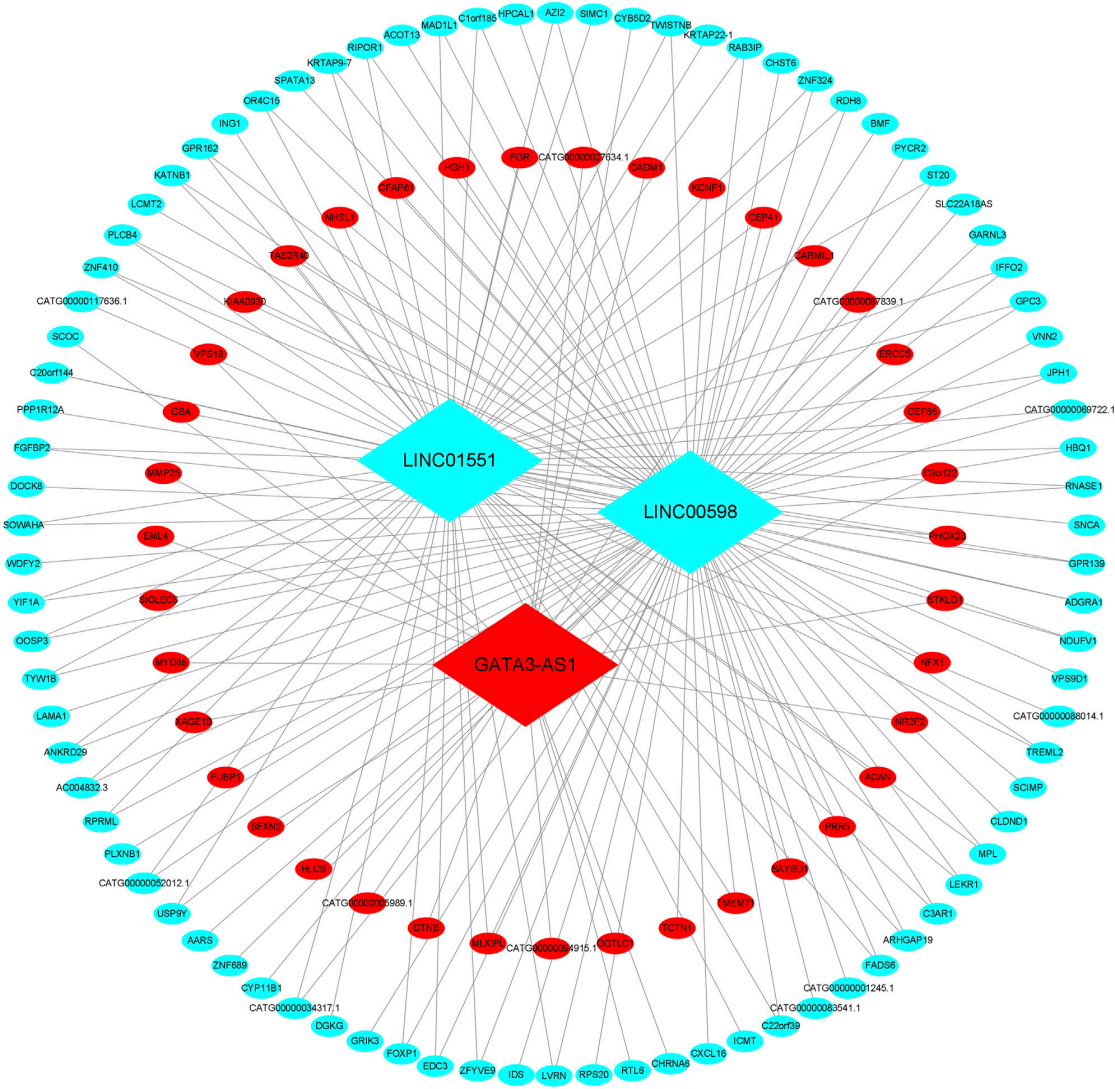
CNC network of the three lncRNAs. The ellipse represents mRNAs, and the diamond represents lncRNAs. Red and green colors represent up- and downregulated RNAs, respectively. CNC, coding–non-coding gene co-expression.

Among the co-expression, three lncRNAs and 125 mRNAs comprise the CNC network, which includes 178 network pairs of co-expressing lncRNAs and mRNAs. As shown in Figure 2, one hub lncRNA can be correlated with many mRNAs, and most of these relational pairs are presented as positive correlations.

### 3.4 GO and KEGG pathway analyses of mRNAs in the CNC network

**FIGURE 3:**
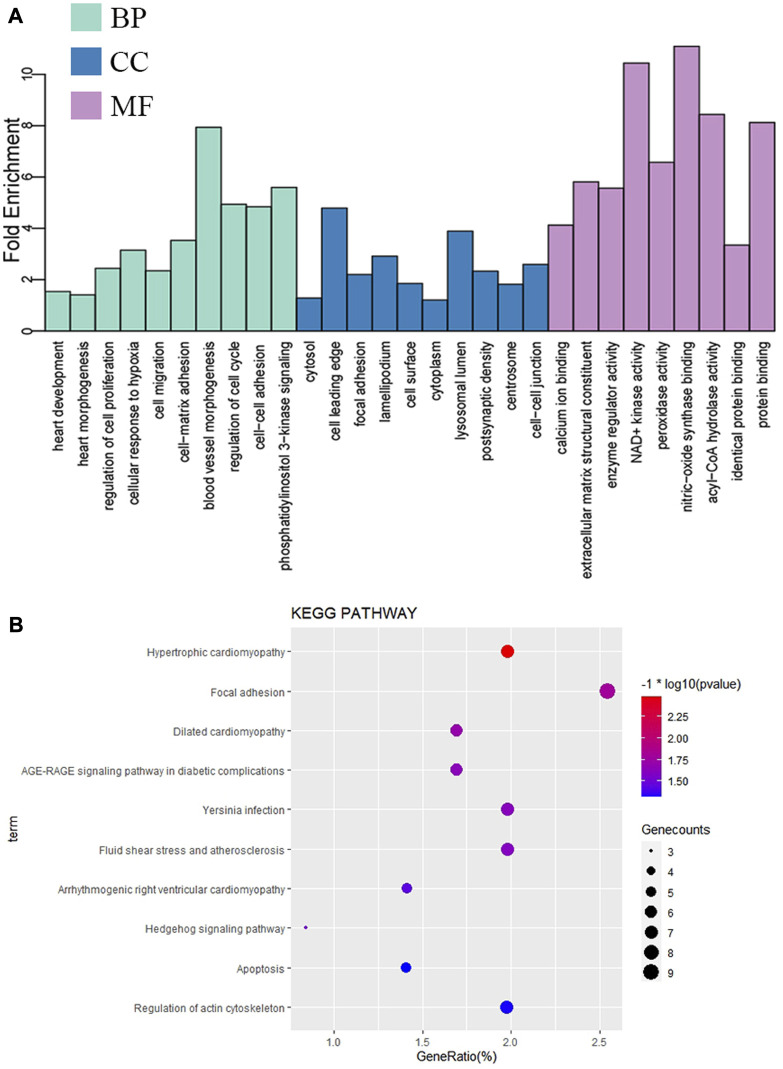
Enrichment analysis of significant DE-mRNAs in VSD. **(A)** Significantly enriched top ten GO terms. **(B)** Significantly enriched top ten KEGG pathways. GO, Gene Ontology; BP, biological process; CC, cellular component; MF, molecular function; KEGG, Kyoto Encyclopedia of Genes and Genomes.

A total of 279 GO terms, including biological processes, molecular functions, and cellular components, were significantly enriched. The top ten terms of the GO results are shown in Figure 3A. Briefly, most genes were enriched in cardiac morphogenesis, cell proliferation, cell cycle, cell–cell junction, and protein binding. The KEGG pathway analysis showed that the mRNAs were enriched in the Hedgehog signaling (Shh) pathway, apoptosis, cardiovascular diseases, and regulation of the actin cytoskeleton. The top ten enriched KEGG pathways are shown in Figure 3B.

### 3.5 WGCNA and identification of VSD-related mRNAs

**FIGURE 4:**
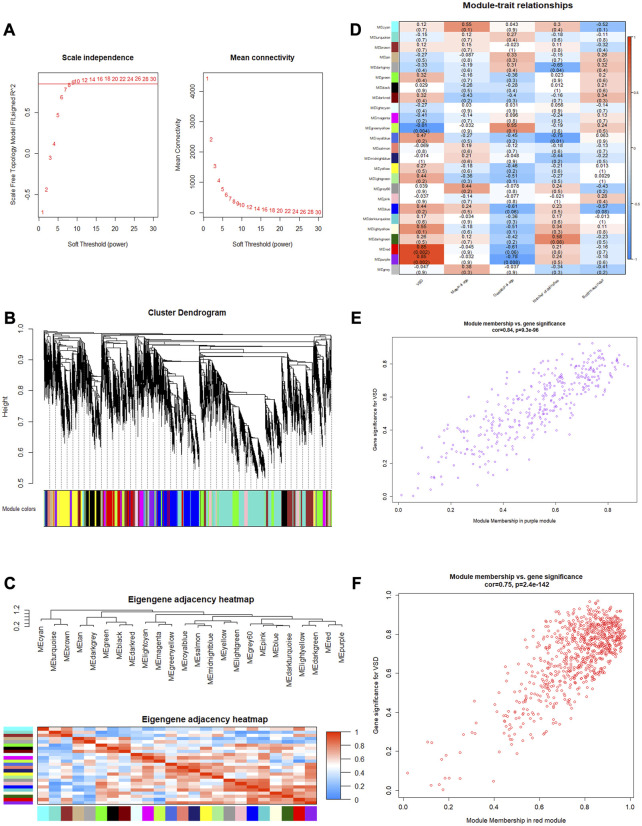
Weighted gene co-expression network analysis (WGCNA). **(A)** Determination of the soft threshold. Left: scale independence; right: mean connectivity. **(B)** Cluster dendrogram. The branches (at the top) represent genes, and each color represents one specific co-expression module. **(C)** Eigengene adjacency heatmap. The color blocks on the left and bottom of the panel correspond to modules. The red color indicates a remarkable correlation between modules. **(D)** Module–trait associations. The columns and rows correspond to trait and module eigengenes, respectively. The red color indicates a positive correlation, and the blue color indicates a negative correlation. **(E, F)** Scatterplots of MM vs. GS in the purple and red modules. GS, gene significance; MM, module membership.

To identify the relatively balanced mean connectivity and scale independence, soft-thresholding power β was set to 9, and the scale-free topology R^2^-value was set to 0.85 (Figure 4A). A total of 24 scale-free topology modules were constructed with the one-step network construction method (Figure 4B). The largest module was turquoise with 2,472 mRNAs, and the smallest module was dark gray with 32 mRNAs. The eigengene adjacency heatmap showed that modules were independent of each other (Figure 4C). The module–trait relationships are shown in Figure 4D. Among these modules, the red and purple modules were most positively associated with VSD. Therefore, we chose the red and purple modules as the key modules of VSD. Plots were charted to show the relationship between MM and GS in the red and purple modules (Figures 4E, F). Under the criteria of GS > 0.3 and MM > 0.8, 408 interesting mRNAs and 25 interesting mRNAs were singled out from the red and purple modules, respectively. After an integrated analysis of module genes and CNC network genes, 16 overlapping mRNAs were identified for further analysis (Supplementary Table S7).

### 3.6 Construction of the ceRNA network

**FIGURE 5:**
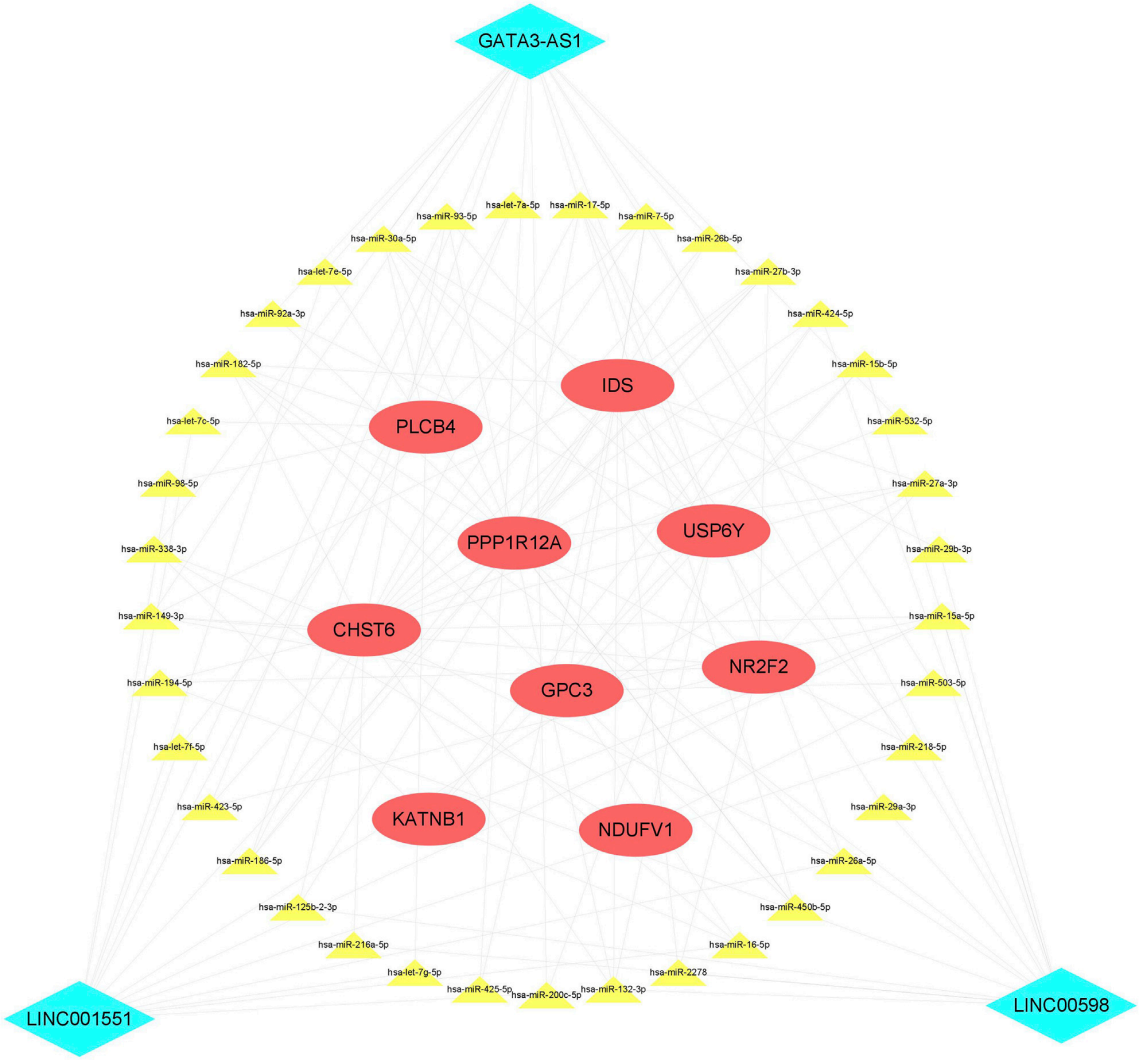
ceRNA network. The red ellipse represents mRNAs, the green diamond represents lncRNAs, and the yellow triangle represents miRNAs. ceRNA, competitive endogenous RNA.

To the best of our knowledge, lncRNAs are positively correlated with mRNAs in the ceRNA regulatory network. Based on the CNC network, 14 positive lncRNA–mRNA pairs, including three lncRNAs and nine mRNAs, were selected to construct the ceRNA. Subsequently, a total of 37 miRNAs were identified from the miRcode and TargetScan databases, exhibiting overlapping target sites on both lncRNAs and mRNAs. Eventually, the ceRNA regulatory network, which includes three lncRNAs, 38 miRNAs, and nine mRNAs, was constructed according to the pairs of lncRNA–mRNA, miRNA–mRNA, and lncRNA–mRNA (Figure 5).

## 4 Discussion

As we all know, cardiac development involves many signaling pathways and is regulated by ncRNAs and mRNAs both temporally and spatially. It has been shown that amniotic fluid remains in constant exchange with maternal blood by the placenta (Rosner et al., 2021), and cell-free fetal RNAs derived from fetal tissues were remarkably stable in peripheral blood (Liu et al., 2022). To explore whether the expression of VSD-related lncRNAs is consistent in maternal plasma and amniotic fluid, we selected four lncRNAs validated in our previous study and performed qPCR in plasma samples. The result showed that three lncRNAs (LINC01551, LINC00598, and GATA3-AS1) were consistent with amniotic fluid. Subsequently, the ROC curves showed that the three lncRNAs were able to significantly differentiate VSD patients from controls. These data indicate that cell-free fetal lncRNAs are capable of bidirectional transport between amniotic fluid and maternal plasma and are expected to be useful biomarkers for VSD diagnosis.

How lncRNAs coordinate to control cell fate remains largely elusive. One possibility is that lncRNAs regulate specific gene expression programs to influence cell function. In the combined analysis of the CNC network and functional enrichment, we found that mRNAs associated with these three lncRNAs were enriched in heart morphogenesis, actin cytoskeleton regulation, cell cycle, and protein binding. Abnormal heart morphogenesis is at the root of CHDs, and the potential CHD may arise at each heart development stage (Baban et al., 2022). For example, the secondary heart field is very important for the outflow tract and chamber formation and, when disrupted, will lead to conotruncal defects (Houyel and Meilhac, 2021). The actin cytoskeleton is pivotal to the proper function of cardiac muscle and the deployment process of second heart field progenitor cells (Wu et al., 2017). The correct cardiogenic lineage cell cycle directs cardiomyocyte reprogramming and stem cell differentiation (Hubert et al., 2018).

As previously discussed, the biological activity of these mRNAs contributes to the differentiation of specific cell types and morphogenesis of critical layers in the developing heart, but the key mRNA for VSD remained unclear. Through WGCNA, the red and purple modules were identified as positively correlated with VSD, and then 16 mRNAs were identified as associated with VSD pathogenesis. Several studies have reported that these mRNAs may cause perturbations in the cardiac structure and function by interfering with cell-type differentiation and specification. In mammals, NR2F2 is abundantly expressed in the developing fetal heart and is considered a candidate gene contributing to CHD (Qiao et al., 2018; Wang et al., 2019). KATNB1 is a critical regulator of heart development, and mutations in KATNB1 will cause heart left–right asymmetry defects (Furtado et al., 2017). GPC3 is widely expressed in embryonic mesodermal tissues and plays an important role in regulating cell proliferation and apoptosis in the embryonic heart (Liu et al., 2021). IDS contributes to cardiomyocyte differentiation disorder and aberrant heart development by affecting the catabolism of GAGs (Matsuhisa and Imaizumi, 2021).

The sponging effect is a particular feature of lncRNAs, which can regulate mRNA by competitively binding to shared miRNAs (Marques and Gama-carvalho, 2022). Therefore, knowledge of the lncRNA-associated ceRNA regulation network and its patterning is central to the understanding of CHD diagnosis and etiology. To reveal the underlying regulatory network between lncRNAs and mRNAs, we constructed a ceRNA network with three lncRNAs, 38 miRNAs, and nine mRNAs. In the ceRNA network, many miRNAs have been confirmed to be involved in the development of CHDs. For example, previous research has reported that has-let-7e (Li et al., 2014), has-miR-27b (Long et al., 2020), and has-miR-218 (Ramachandran et al., 2022) were dysregulated in CHD patients. In addition, Yang et al. (2020) found that has-miR-29b was significantly overexpressed in the right ventricular outflow tract of CHD patients and inhibited cardiomyocyte proliferation by targeting NOTCH2. Liu et al. (2016) found that has-miR-30c was upregulated in the heart tissues with VSD and confirmed that miR-30c regulated P19 cell differentiation, apoptosis, and proliferation via the Hedgehog signaling pathway. The aforementioned studies show that an lncRNA can sponge off one or more miRNAs to affect downstream target gene expression and participate in the formation of VSD.

## 5 Conclusion

In the present study, we confirmed that several VSD-related lncRNAs (GATA3-AS1, LINC01551, and LINC00598) were consistent in both maternal plasma and amniotic fluid, suggesting that the amniotic fluid cell-free lncRNAs could be released into the maternal circulation and investigated as potential markers for CHDs. The effects of these lncRNAs on VSD may be involved in cardiac morphogenesis, actin cytoskeleton, cell cycle, and protein binding by the ceRNA network at the post-transcriptional level. Nevertheless, our study has several limitations. First, the sample size enrolled in the present study is insufficient. In the future, larger samples with VSD are needed to validate those candidate lncRNAs. Second, we have only predicted the potential role of lncRNAs in the development of VSD through bioinformatics analysis, but the targets and specific mechanisms of these lncRNAs have not been validated. Therefore, the corresponding functional and mechanistic experiments need to be initiated urgently *in vivo* and *in vitro*.

## Data Availability

Publicly available datasets were analyzed in this study. These data can be found at: Gene Expression Omnibus (GEO) site (https://www.ncbi.nlm.nih.gov/geo/) (GEO: GSE204935).
