# Supplementary material for: Maternal body fluid lncRNAs serve as biomarkers to diagnose ventricular septal defect: from amniotic fluid to plasma
Source: Front Genet. 2023 Sep 6;14:1254829. doi: 10.3389/fgene.2023.1254829 (PMC10516564; doi:10.3389/fgene.2023.1254829)
Supplement: Supplementary file 1 [file DataSheet1.docx]

Table S1 Sequences of the primer used for qPCR

| Gene | Forward primer (5'-3') | Reverse primer (3'-5') |
| --- | --- | --- |
| LINC00598 | ATCTTCCTCCTCCTCACTTCTCTGC | GGCTTCTCTGGTTATCACTCGTTCC |
| GATA3-AS1 | TCTTGGGAAAAAGGCAGAAGAAAG | TTTCTGGCCTTTGGTGTCGC |
| PWRN1 | AAAACATCTCGGCAGGTAAAG | GCAATCAGACACACAAGCACA |
| LINC01551 | GCAGACAGAAGCCCTTGAAGT | TCAACACTGTCCACTACCCCTA |
| GAPDH | TTGGTATCGTGGAAGGACTCA | AGTAGAGGCAGGGATGATGTT |

Table S2 Detailed clinical data for each individual human subject

| Sample | Maternal age (years) | Gestational age (weeks) | Number of deliveries | Body mass index (kg/m^2^) |
| --- | --- | --- | --- | --- |
| T1 | 31 | 22 | 2 | 23 |
| T2 | 34 | 20 | 2 | 21 |
| T3 | 24 | 20 | 1 | 22 |
| T4 | 31 | 20 | 3 | 21 |
| T5 | 37 | 22 | 3 | 22 |
| T6 | 35 | 22 | 2 | 23 |
| T7 | 26 | 21 | 1 | 21 |
| T8 | 29 | 22 | 1 | 24 |
| T9 | 34 | 21 | 3 | 23 |
| T10 | 29 | 22 | 2 | 21 |
| T11 | 29 | 21 | 1 | 22 |
| T12 | 27 | 20 | 2 | 21 |
| T13 | 28 | 20 | 3 | 22 |
| T14 | 30 | 22 | 3 | 22 |
| T15 | 31 | 21 | 2 | 21 |
| T16 | 27 | 22 | 1 | 24 |
| T17 | 29 | 22 | 1 | 21 |
| T18 | 30 | 22 | 2 | 21 |
| T19 | 28 | 20 | 2 | 21 |
| T20 | 31 | 20 | 1 | 24 |
| T21 | 29 | 22 | 2 | 23 |
| T22 | 30 | 21 | 1 | 21 |
| N1 | 33 | 21 | 2 | 24 |
| N2 | 33 | 21 | 3 | 19 |
| N3 | 34 | 20 | 2 | 22 |
| N4 | 33 | 22 | 1 | 23 |
| N5 | 35 | 20 | 2 | 21 |
| N6 | 30 | 21 | 3 | 22 |
| N7 | 27 | 20 | 1 | 22 |
| N8 | 31 | 20 | 2 | 21 |
| N9 | 19 | 22 | 1 | 23 |
| N10 | 34 | 20 | 3 | 22 |
| N11 | 39 | 20 | 3 | 21 |
| N12 | 32 | 21 | 2 | 22 |
| N13 | 27 | 21 | 2 | 22 |
| N14 | 28 | 20 | 2 | 21 |
| N15 | 29 | 22 | 3 | 23 |
| N16 | 28 | 20 | 2 | 22 |

Table S3 RNA quantification and quality assessment by NanoDrop ND-1000

| Sample | Conc. (ng/μl) | Volume (μl) | Quantity (ng) | OD260/280 Ratio | OD260/230 Ratio |
| --- | --- | --- | --- | --- | --- |
| T1 | 56.20 | 15 | 843.00 | 1.75 | 0.50 |
| T2 | 74.31 | 15 | 1114.65 | 1.84 | 0.44 |
| T3 | 73.07 | 15 | 1096.05 | 1.85 | 0.72 |
| T4 | 98.26 | 15 | 1473.90 | 1.76 | 0.65 |
| T5 | 69.13 | 15 | 1036.95 | 1.79 | 0.89 |
| T6 | 71.99 | 15 | 1079.85 | 1.79 | 0.60 |
| T7 | 45.35 | 15 | 680.25 | 1.87 | 0.72 |
| T8 | 102.63 | 15 | 1539.45 | 1.77 | 0.44 |
| T9 | 121.00 | 15 | 1817.10 | 1.94 | 0.79 |
| T10 | 53.63 | 15 | 804.45 | 1.79 | 0.55 |
| T11 | 96.50 | 15 | 1447.50 | 1.92 | 0.55 |
| T12 | 104.51 | 15 | 1567.65 | 1.85 | 0.65 |
| T13 | 69.18 | 15 | 1037.70 | 1.90 | 0.48 |
| T14 | 51.47 | 15 | 772.05 | 1.74 | 0.56 |
| T15 | 57.63 | 15 | 864.45 | 1.62 | 0.53 |
| T16 | 56.73 | 15 | 850.95 | 1.69 | 0.61 |
| T17 | 75.25 | 15 | 1128.75 | 1.78 | 0.64 |
| T18 | 68.98 | 15 | 1034.70 | 1.81 | 0.71 |
| T19 | 85.35 | 15 | 1280.25 | 1.83 | 0.53 |
| T20 | 58.41 | 15 | 876.15 | 1.76 | 0.58 |
| T21 | 79.35 | 15 | 1190.25 | 1.91 | 0.67 |
| T22 | 101.25 | 15 | 1518.75 | 1.75 | 0.73 |
| N1 | 97.35 | 15 | 1460.25 | 1.88 | 0.71 |
| N2 | 84.53 | 15 | 1267.95 | 1.67 | 0.82 |
| N3 | 78.65 | 15 | 1179.75 | 1.73 | 0.59 |
| N4 | 104.29 | 15 | 1564.35 | 1.86 | 0.67 |
| N5 | 68.24 | 15 | 1023.60 | 1.76 | 0.74 |
| N6 | 77.84 | 15 | 1167.60 | 1.77 | 0.66 |
| N7 | 92.33 | 15 | 1384.95 | 1.85 | 0.84 |
| N8 | 82.52 | 15 | 1237.80 | 1.68 | 0.71 |
| N9 | 93.15 | 15 | 1397.25 | 1.74 | 0.59 |
| N10 | 86.49 | 15 | 1297.35 | 1.73 | 0.63 |
| N11 | 81.32 | 15 | 1219.80 | 1.82 | 0.67 |
| N12 | 59.72 | 15 | 895.80 | 1.90 | 0.82 |
| N13 | 63.54 | 15 | 953.10 | 1.87 | 0.78 |
| N14 | 106.15 | 15 | 1592.25 | 1.74 | 0.74 |
| N15 | 94.51 | 15 | 1417.65 | 1.83 | 0.64 |
| N16 | 76.15 | 15 | 1142.25 | 1.79 | 0.77 |

Table S4 The qPCR data of LINC00598

| Sample Name | LINC00598 | GAPDH | SD | average | △CT | △△CT | 2^^△△CT^ |
| --- | --- | --- | --- | --- | --- | --- | --- |
| T1 | 32.651 | 28.632 | 0.515 | 32.651 | 4.019 | -0.387 | 0.765 |
| T1 | 33.167 | 28.028 | 0.604 | 28.632 |  |  |  |
| T1 | 32.136 | 29.236 |  |  |  |  |  |
| T2 | 33.163 | 28.092 | 0.620 | 33.783 | 5.735 | -2.103 | 0.233 |
| T2 | 33.783 | 27.984 | 0.057 | 28.048 |  |  |  |
| T2 | 34.404 | 28.068 |  |  |  |  |  |
| T3 | 30.842 | 24.515 | 0.149 | 30.822 | 6.231 | -2.599 | 0.165 |
| T3 | 30.663 | 24.823 | 0.205 | 24.590 |  |  |  |
| T3 | 30.960 | 24.433 |  |  |  |  |  |
| T4 | 30.748 | 27.300 | 0.682 | 31.035 | 3.795 | -0.162 | 0.894 |
| T4 | 31.813 | 27.194 | 0.054 | 27.240 |  |  |  |
| T4 | 30.543 | 27.226 |  |  |  |  |  |
| T5 | 33.369 | 28.168 | 0.800 | 32.471 | 4.668 | -1.035 | 0.488 |
| T5 | 32.209 | 27.256 | 0.483 | 27.803 |  |  |  |
| T5 | 31.835 | 27.985 |  |  |  |  |  |
| T6 | 32.564 | 26.568 | 0.685 | 31.910 | 4.833 | -1.200 | 0.435 |
| T6 | 31.198 | 27.462 | 0.460 | 27.077 |  |  |  |
| T6 | 31.969 | 27.202 |  |  |  |  |  |
| T7 | 30.153 | 27.209 | 1.038 | 30.680 | 3.371 | 0.262 | 1.199 |
| T7 | 31.875 | 27.461 | 0.134 | 27.309 |  |  |  |
| T7 | 30.011 | 27.257 |  |  |  |  |  |
| T8 | 35.251 | 26.704 | 1.049 | 34.081 | 6.826 | -3.194 | 0.109 |
| T8 | 33.224 | 27.255 | 0.551 | 27.255 |  |  |  |
| T8 | 33.769 | 27.806 |  |  |  |  |  |
| T9 | 32.756 | 26.045 | 5.431 | 29.983 | 3.999 | -0.366 | 0.776 |
| T9 | 23.726 | 25.857 | 0.110 | 25.985 |  |  |  |
| T9 | 33.468 | 26.051 |  |  |  |  |  |
| T10 | 37.125 | 26.051 | 2.222 | 34.903 | 7.887 | -4.254 | 0.052 |
| T10 | 32.681 | 27.980 | 0.964 | 27.015 |  |  |  |
| T10 | 34.902 | 27.015 |  |  |  |  |  |
| T11 | 29.641 | 25.805 | 1.587 | 30.467 | 4.632 | -0.999 | 0.500 |
| T11 | 32.297 | 25.724 | 0.129 | 25.835 |  |  |  |
| T11 | 29.463 | 25.977 |  |  |  |  |  |
| T12 | 34.268 | 27.575 | 0.561 | 34.732 | 6.886 | -3.254 | 0.105 |
| T12 | 34.572 | 27.980 | 0.234 | 27.845 |  |  |  |
| T12 | 35.356 | 27.982 |  |  |  |  |  |
| T13 | 35.647 | 28.894 | 1.812 | 33.714 | 5.509 | -1.876 | 0.272 |
| T13 | 32.053 | 28.026 | 0.618 | 28.205 |  |  |  |
| T13 | 33.443 | 27.697 |  |  |  |  |  |
| T14 | 31.986 | 28.023 | 1.318 | 33.105 | 5.009 | -1.376 | 0.385 |
| T14 | 32.771 | 27.945 | 0.198 | 28.096 |  |  |  |
| T14 | 34.558 | 28.320 |  |  |  |  |  |
| T15 | 35.803 | 28.313 | 3.440 | 31.916 | 3.331 | 0.302 | 1.233 |
| T15 | 30.682 | 28.164 | 0.605 | 28.585 |  |  |  |
| T15 | 29.263 | 29.279 |  |  |  |  |  |
| T16 | 30.786 | 26.731 | 0.177 | 30.608 | 4.177 | -0.544 | 0.686 |
| T16 | 30.431 | 26.291 | 0.260 | 26.431 |  |  |  |
| T16 | 30.608 | 26.272 |  |  |  |  |  |
| T17 | 31.238 | 27.486 | 1.692 | 33.158 | 5.642 | -2.010 | 0.248 |
| T17 | 33.807 | 27.496 | 0.044 | 27.516 |  |  |  |
| T17 | 34.430 | 27.566 |  |  |  |  |  |
| T18 | 31.358 | 25.103 | 0.259 | 31.616 | 6.294 | -2.661 | 0.158 |
| T18 | 31.875 | 25.595 | 0.251 | 25.322 |  |  |  |
| T18 | 31.616 | 25.268 |  |  |  |  |  |
| T19 | 35.059 | 27.395 | 1.062 | 34.338 | 6.389 | -2.756 | 0.148 |
| T19 | 33.119 | 28.628 | 0.626 | 27.949 |  |  |  |
| T19 | 34.837 | 27.825 |  |  |  |  |  |
| T20 | 31.919 | 26.672 | 0.731 | 31.912 | 5.417 | -1.784 | 0.290 |
| T20 | 31.177 | 26.360 | 0.160 | 26.495 |  |  |  |
| T20 | 32.639 | 26.453 |  |  |  |  |  |
| T21 | 30.749 | 25.602 | 0.390 | 30.999 | 5.736 | -2.103 | 0.233 |
| T21 | 30.799 | 25.089 | 0.294 | 25.263 |  |  |  |
| T21 | 31.449 | 25.099 |  |  |  |  |  |
| T22 | 32.251 | 30.654 | 1.666 | 34.147 | 3.638 | -0.006 | 0.996 |
| T22 | 34.818 | 30.571 | 0.184 | 30.509 |  |  |  |
| T22 | 35.374 | 30.302 |  |  |  |  |  |
| N1 | 30.413 | 28.093 | 0.452 | 30.445 | 2.389 | 1.244 | 2.368 |
| N1 | 30.011 | 28.059 | 0.038 | 28.056 |  |  |  |
| N1 | 30.913 | 28.017 |  |  |  |  |  |
| N2 | 29.996 | 25.812 | 0.662 | 30.122 | 4.303 | -0.671 | 0.628 |
| N2 | 30.838 | 25.673 | 0.149 | 25.819 |  |  |  |
| N2 | 29.531 | 25.971 |  |  |  |  |  |
| N3 | 29.727 | 26.631 | 1.314 | 30.357 | 3.720 | -0.087 | 0.941 |
| N3 | 29.477 | 26.650 | 0.011 | 26.637 |  |  |  |
| N3 | 31.868 | 26.631 |  |  |  |  |  |
| N4 | 30.514 | 26.031 | 0.890 | 29.852 | 3.616 | 0.017 | 1.012 |
| N4 | 30.201 | 26.351 | 0.178 | 26.236 |  |  |  |
| N4 | 28.840 | 26.325 |  |  |  |  |  |
| N5 | 35.928 | 31.496 | 2.889 | 34.085 | 3.213 | 0.420 | 1.338 |
| N5 | 30.756 | 30.933 | 0.657 | 30.872 |  |  |  |
| N5 | 35.571 | 30.187 |  |  |  |  |  |
| N6 | 34.480 | 30.232 | 0.215 | 34.695 | 4.238 | -0.605 | 0.657 |
| N6 | 34.911 | 30.683 | 0.226 | 30.458 |  |  |  |
| N6 | 34.695 | 30.457 |  |  |  |  |  |
| N7 | 33.527 | 29.732 | 0.127 | 33.665 | 4.147 | -0.514 | 0.700 |
| N7 | 33.691 | 29.380 | 0.188 | 29.518 |  |  |  |
| N7 | 33.777 | 29.443 |  |  |  |  |  |
| N8 | 30.338 | 27.720 | 0.730 | 29.985 | 2.555 | 1.078 | 2.111 |
| N8 | 30.471 | 27.320 | 0.254 | 27.430 |  |  |  |
| N8 | 29.145 | 27.250 |  |  |  |  |  |
| N9 | 34.773 | 30.258 | 0.428 | 34.345 | 3.727 | -0.094 | 0.937 |
| N9 | 33.917 | 30.618 | 0.360 | 30.618 |  |  |  |
| N9 | 34.345 | 30.978 |  |  |  |  |  |
| N10 | 30.576 | 26.866 | 0.311 | 30.408 | 3.392 | 0.241 | 1.182 |
| N10 | 30.600 | 26.936 | 0.203 | 27.017 |  |  |  |
| N10 | 30.049 | 27.248 |  |  |  |  |  |
| N11 | 35.265 | 29.549 | 2.654 | 33.969 | 3.953 | -0.320 | 0.801 |
| N11 | 35.726 | 30.016 | 0.467 | 30.016 |  |  |  |
| N11 | 30.916 | 30.483 |  |  |  |  |  |
| N12 | 31.531 | 27.187 | 0.122 | 31.461 | 4.320 | -0.687 | 0.621 |
| N12 | 31.320 | 26.942 | 0.181 | 27.142 |  |  |  |
| N12 | 31.533 | 27.296 |  |  |  |  |  |
| N13 | 28.526 | 25.383 | 0.206 | 28.751 | 3.391 | 0.242 | 1.182 |
| N13 | 28.929 | 25.344 | 0.021 | 25.360 |  |  |  |
| N13 | 28.800 | 25.353 |  |  |  |  |  |
| N14 | 30.161 | 27.311 | 0.352 | 30.558 | 3.581 | 0.052 | 1.037 |
| N14 | 30.676 | 26.907 | 0.305 | 26.977 |  |  |  |
| N14 | 30.836 | 26.712 |  |  |  |  |  |
| N15 | 30.717 | 27.725 | 0.046 | 30.764 | 2.951 | 0.682 | 1.604 |
| N15 | 30.810 | 27.983 | 0.147 | 27.813 |  |  |  |
| N15 | 30.763 | 27.731 |  |  |  |  |  |
| N16 | 31.730 | 28.482 | 1.793 | 33.240 | 4.629 | -0.996 | 0.501 |
| N16 | 32.768 | 29.053 | 0.394 | 28.611 |  |  |  |
| N16 | 35.222 | 28.297 |  |  |  |  |  |

Table S5 The qPCR data of LINC001551

| Sample Name | LINC01551 | GAPDH | SD | average | △CT | △△CT | 2^^△△CT^ |
| --- | --- | --- | --- | --- | --- | --- | --- |
| T1 | 34.212 | 30.132 | 1.154 | 34.213 | 4.080 | -2.081 | 0.236 |
| T1 | 35.367 | 30.028 | 0.104 | 30.132 |  |  |  |
| T1 | 33.059 | 30.236 |  |  |  |  |  |
| T2 | 31.646 | 28.092 | 0.339 | 31.646 | 3.598 | -1.599 | 0.330 |
| T2 | 31.985 | 27.984 | 0.057 | 28.048 |  |  |  |
| T2 | 31.307 | 28.068 |  |  |  |  |  |
| T3 | 29.130 | 24.515 | 0.027 | 29.130 | 4.540 | -2.541 | 0.172 |
| T3 | 29.158 | 24.823 | 0.205 | 24.590 |  |  |  |
| T3 | 29.103 | 24.433 |  |  |  |  |  |
| T4 | 24.264 | 27.300 | 6.505 | 31.132 | 3.893 | -1.893 | 0.269 |
| T4 | 31.933 | 27.194 | 0.054 | 27.240 |  |  |  |
| T4 | 37.200 | 27.226 |  |  |  |  |  |
| T5 | 30.520 | 27.168 | 0.335 | 30.520 | 3.075 | -1.076 | 0.474 |
| T5 | 30.855 | 27.571 | 0.240 | 27.445 |  |  |  |
| T5 | 30.185 | 27.596 |  |  |  |  |  |
| T6 | 31.337 | 30.463 | 1.194 | 32.530 | 2.067 | -0.068 | 0.954 |
| T6 | 32.530 | 30.725 | 0.262 | 30.463 |  |  |  |
| T6 | 33.724 | 30.202 |  |  |  |  |  |
| T7 | 31.764 | 27.209 | 0.069 | 31.695 | 4.386 | -2.387 | 0.191 |
| T7 | 31.627 | 27.461 | 0.134 | 27.309 |  |  |  |
| T7 | 31.695 | 27.257 |  |  |  |  |  |
| T8 | 37.841 | 32.704 | 3.404 | 36.197 | 2.442 | -0.443 | 0.735 |
| T8 | 32.283 | 33.755 | 1.051 | 33.755 |  |  |  |
| T8 | 38.468 | 34.806 |  |  |  |  |  |
| T9 | 29.456 | 26.045 | 1.202 | 30.658 | 4.673 | -2.674 | 0.157 |
| T9 | 31.860 | 25.857 | 0.110 | 25.985 |  |  |  |
| T9 | 30.657 | 26.051 |  |  |  |  |  |
| T10 | 33.124 | 29.571 | 0.819 | 33.124 | 3.444 | -1.445 | 0.367 |
| T10 | 32.306 | 29.789 | 0.109 | 29.680 |  |  |  |
| T10 | 33.943 | 29.680 |  |  |  |  |  |
| T11 | 30.156 | 25.805 | 0.941 | 30.109 | 4.274 | -2.274 | 0.207 |
| T11 | 31.025 | 25.724 | 0.129 | 25.835 |  |  |  |
| T11 | 29.145 | 25.977 |  |  |  |  |  |
| T12 | 33.014 | 27.575 | 1.043 | 32.650 | 4.805 | -2.805 | 0.143 |
| T12 | 33.462 | 27.980 | 0.234 | 27.845 |  |  |  |
| T12 | 31.474 | 27.982 |  |  |  |  |  |
| T13 | 31.138 | 28.894 | 0.092 | 31.230 | 3.025 | -1.026 | 0.491 |
| T13 | 31.230 | 28.026 | 0.618 | 28.205 |  |  |  |
| T13 | 31.323 | 27.697 |  |  |  |  |  |
| T14 | 31.935 | 28.023 | 1.101 | 31.935 | 3.839 | -1.840 | 0.279 |
| T14 | 33.036 | 27.945 | 0.198 | 28.096 |  |  |  |
| T14 | 30.835 | 28.320 |  |  |  |  |  |
| T15 | 30.712 | 28.268 | 0.722 | 30.989 | 2.752 | -0.753 | 0.593 |
| T15 | 31.809 | 28.164 | 0.064 | 28.237 |  |  |  |
| T15 | 30.446 | 28.279 |  |  |  |  |  |
| T16 | 28.453 | 26.731 | 1.410 | 29.863 | 3.431 | -1.432 | 0.371 |
| T16 | 29.863 | 26.291 | 0.260 | 26.431 |  |  |  |
| T16 | 31.272 | 26.272 |  |  |  |  |  |
| T17 | 31.689 | 27.486 | 1.973 | 31.725 | 4.209 | -2.210 | 0.216 |
| T17 | 33.716 | 27.496 | 0.044 | 27.516 |  |  |  |
| T17 | 29.771 | 27.566 |  |  |  |  |  |
| T18 | 30.555 | 25.103 | 0.576 | 30.100 | 4.778 | -2.779 | 0.146 |
| T18 | 29.453 | 25.595 | 0.251 | 25.322 |  |  |  |
| T18 | 30.293 | 25.268 |  |  |  |  |  |
| T19 | 29.665 | 27.395 | 1.023 | 30.368 | 2.419 | -0.420 | 0.748 |
| T19 | 31.542 | 28.628 | 0.626 | 27.949 |  |  |  |
| T19 | 29.897 | 27.825 |  |  |  |  |  |
| T20 | 30.531 | 26.672 | 0.192 | 30.531 | 4.036 | -2.037 | 0.244 |
| T20 | 30.723 | 26.360 | 0.160 | 26.495 |  |  |  |
| T20 | 30.339 | 26.453 |  |  |  |  |  |
| T21 | 30.395 | 25.602 | 0.102 | 30.399 | 5.135 | -3.136 | 0.114 |
| T21 | 30.502 | 25.089 | 0.294 | 25.263 |  |  |  |
| T21 | 30.299 | 25.099 |  |  |  |  |  |
| T22 | 35.412 | 30.654 | 1.478 | 34.050 | 3.541 | -1.542 | 0.343 |
| T22 | 32.479 | 30.571 | 0.184 | 30.509 |  |  |  |
| T22 | 34.259 | 30.302 |  |  |  |  |  |
| N1 | 29.556 | 28.093 | 0.132 | 29.688 | 1.631 | 0.368 | 1.290 |
| N1 | 29.687 | 28.059 | 0.038 | 28.056 |  |  |  |
| N1 | 29.819 | 28.017 |  |  |  |  |  |
| N2 | 27.672 | 25.812 | 0.513 | 28.142 | 2.323 | -0.324 | 0.799 |
| N2 | 28.689 | 25.673 | 0.149 | 25.819 |  |  |  |
| N2 | 28.064 | 25.971 |  |  |  |  |  |
| N3 | 29.700 | 26.631 | 0.146 | 29.555 | 2.917 | -0.918 | 0.529 |
| N3 | 29.408 | 26.650 | 0.011 | 26.637 |  |  |  |
| N3 | 29.556 | 26.631 |  |  |  |  |  |
| N4 | 28.765 | 26.031 | 0.251 | 29.005 | 2.769 | -0.770 | 0.586 |
| N4 | 28.985 | 26.351 | 0.178 | 26.236 |  |  |  |
| N4 | 29.266 | 26.325 |  |  |  |  |  |
| N5 | 30.452 | 29.496 | 0.236 | 30.452 | 1.247 | 0.752 | 1.684 |
| N5 | 30.688 | 29.933 | 0.909 | 29.205 |  |  |  |
| N5 | 30.217 | 28.187 |  |  |  |  |  |
| N6 | 30.581 | 28.232 | 0.419 | 30.327 | 1.870 | 0.130 | 1.094 |
| N6 | 29.843 | 28.683 | 0.226 | 28.458 |  |  |  |
| N6 | 30.558 | 28.457 |  |  |  |  |  |
| N7 | 31.876 | 29.732 | 0.479 | 31.876 | 2.358 | -0.359 | 0.780 |
| N7 | 32.356 | 29.380 | 0.188 | 29.518 |  |  |  |
| N7 | 31.397 | 29.443 |  |  |  |  |  |
| N8 | 30.278 | 27.720 | 0.748 | 29.564 | 2.133 | -0.134 | 0.911 |
| N8 | 29.627 | 27.320 | 0.254 | 27.430 |  |  |  |
| N8 | 28.785 | 27.250 |  |  |  |  |  |
| N9 | 31.701 | 30.258 | 1.678 | 31.955 | 1.337 | 0.662 | 1.582 |
| N9 | 33.745 | 30.618 | 0.360 | 30.618 |  |  |  |
| N9 | 30.419 | 30.978 |  |  |  |  |  |
| N10 | 28.756 | 26.866 | 0.070 | 28.680 | 1.664 | 0.336 | 1.262 |
| N10 | 28.617 | 26.936 | 0.203 | 27.017 |  |  |  |
| N10 | 28.667 | 27.248 |  |  |  |  |  |
| N11 | 31.648 | 29.549 | 0.141 | 31.648 | 1.519 | 0.480 | 1.395 |
| N11 | 31.789 | 30.356 | 0.506 | 30.129 |  |  |  |
| N11 | 31.507 | 30.483 |  |  |  |  |  |
| N12 | 30.163 | 27.187 | 0.147 | 30.163 | 3.021 | -1.022 | 0.492 |
| N12 | 30.310 | 26.942 | 0.181 | 27.142 |  |  |  |
| N12 | 30.016 | 27.296 |  |  |  |  |  |
| N13 | 28.135 | 25.383 | 0.872 | 27.263 | 1.903 | 0.096 | 1.069 |
| N13 | 26.391 | 25.344 | 0.021 | 25.360 |  |  |  |
| N13 | 27.264 | 25.353 |  |  |  |  |  |
| N14 | 28.794 | 27.311 | 0.791 | 29.002 | 2.026 | -0.026 | 0.982 |
| N14 | 28.337 | 26.907 | 0.305 | 26.977 |  |  |  |
| N14 | 29.877 | 26.712 |  |  |  |  |  |
| N15 | 29.358 | 27.725 | 0.195 | 29.195 | 1.382 | 0.617 | 1.533 |
| N15 | 29.248 | 27.983 | 0.147 | 27.813 |  |  |  |
| N15 | 28.980 | 27.731 |  |  |  |  |  |
| N16 | 30.502 | 28.482 | 0.005 | 30.497 | 1.886 | 0.113 | 1.081 |
| N16 | 30.492 | 29.053 | 0.394 | 28.611 |  |  |  |
| N16 | 30.497 | 28.297 |  |  |  |  |  |

Table S6 The qPCR data of GATA3-AS1

| Sample Name | GATA3-AS1 | GAPDH | SD | average | △CT | △△CT | 2^^△△CT^ |
| --- | --- | --- | --- | --- | --- | --- | --- |
| T1 | 30.565 | 28.549 | 0.372 | 30.142 | 2.419 | 1.835 | 3.568 |
| T1 | 29.995 | 27.364 | 0.717 | 27.723 |  |  |  |
| T1 | 29.865 | 27.256 |  |  |  |  |  |
| T2 | 30.031 | 28.092 | 0.064 | 30.101 | 2.053 | 2.201 | 4.598 |
| T2 | 30.114 | 27.984 | 0.057 | 28.048 |  |  |  |
| T2 | 30.157 | 28.068 |  |  |  |  |  |
| T3 | 25.098 | 24.475 | 1.252 | 26.272 | 1.941 | 2.312 | 4.967 |
| T3 | 27.589 | 24.221 | 0.130 | 24.331 |  |  |  |
| T3 | 26.130 | 24.296 |  |  |  |  |  |
| T4 | 27.183 | 26.328 | 1.321 | 28.500 | 2.070 | 2.184 | 4.543 |
| T4 | 29.825 | 27.152 | 0.677 | 26.430 |  |  |  |
| T4 | 28.492 | 25.809 |  |  |  |  |  |
| T5 | 30.311 | 28.695 | 0.565 | 30.143 | 1.729 | 2.525 | 5.754 |
| T5 | 30.604 | 28.133 | 0.281 | 28.414 |  |  |  |
| T5 | 29.513 | 28.413 |  |  |  |  |  |
| T6 | 29.863 | 27.428 | 0.429 | 30.291 | 2.725 | 1.529 | 2.886 |
| T6 | 30.288 | 27.705 | 0.139 | 27.566 |  |  |  |
| T6 | 30.722 | 27.566 |  |  |  |  |  |
| T7 | 30.648 | 27.820 | 0.513 | 31.079 | 3.990 | 0.264 | 1.201 |
| T7 | 30.941 | 26.358 | 0.731 | 27.089 |  |  |  |
| T7 | 31.647 | 27.089 |  |  |  |  |  |
| T8 | 31.208 | 26.924 | 1.695 | 31.284 | 4.153 | 0.101 | 1.073 |
| T8 | 33.016 | 26.967 | 0.323 | 27.132 |  |  |  |
| T8 | 29.628 | 27.504 |  |  |  |  |  |
| T9 | 27.794 | 25.916 | 2.040 | 28.557 | 2.948 | 1.306 | 2.473 |
| T9 | 27.008 | 25.309 | 0.304 | 25.609 |  |  |  |
| T9 | 30.869 | 25.603 |  |  |  |  |  |
| T10 | 29.749 | 27.385 | 0.464 | 29.542 | 2.156 | 2.097 | 4.280 |
| T10 | 29.866 | 27.959 | 0.574 | 27.385 |  |  |  |
| T10 | 29.010 | 26.812 |  |  |  |  |  |
| T11 | 29.563 | 25.805 | 0.493 | 29.630 | 3.794 | 0.459 | 1.375 |
| T11 | 29.174 | 25.724 | 0.129 | 25.835 |  |  |  |
| T11 | 30.152 | 25.977 |  |  |  |  |  |
| T12 | 29.565 | 27.317 | 0.709 | 30.381 | 3.043 | 1.211 | 2.314 |
| T12 | 30.732 | 27.678 | 0.331 | 27.337 |  |  |  |
| T12 | 30.845 | 27.017 |  |  |  |  |  |
| T13 | 31.970 | 28.894 | 0.766 | 31.674 | 3.469 | 0.785 | 1.723 |
| T13 | 30.804 | 28.026 | 0.618 | 28.205 |  |  |  |
| T13 | 32.249 | 27.697 |  |  |  |  |  |
| T14 | 30.468 | 29.269 | 0.893 | 31.397 | 2.669 | 1.585 | 2.999 |
| T14 | 31.474 | 28.840 | 0.605 | 28.728 |  |  |  |
| T14 | 32.249 | 28.074 |  |  |  |  |  |
| T15 | 26.138 | 27.016 | 2.426 | 28.911 | 1.758 | 2.495 | 5.639 |
| T15 | 29.954 | 27.379 | 0.198 | 27.152 |  |  |  |
| T15 | 30.640 | 27.062 |  |  |  |  |  |
| T16 | 29.460 | 25.897 | 1.124 | 30.559 | 3.841 | 0.413 | 1.331 |
| T16 | 31.707 | 28.705 | 1.730 | 26.718 |  |  |  |
| T16 | 30.509 | 25.551 |  |  |  |  |  |
| T17 | 29.938 | 28.602 | 1.230 | 30.479 | 2.429 | 1.825 | 3.543 |
| T17 | 29.613 | 27.815 | 0.479 | 28.051 |  |  |  |
| T17 | 31.888 | 27.736 |  |  |  |  |  |
| T18 | 29.310 | 25.397 | 0.166 | 29.482 | 4.187 | 0.067 | 1.048 |
| T18 | 29.494 | 25.321 | 0.116 | 25.296 |  |  |  |
| T18 | 29.642 | 25.169 |  |  |  |  |  |
| T19 | 31.490 | 27.155 | 0.125 | 31.522 | 4.311 | -0.057 | 0.961 |
| T19 | 31.660 | 27.303 | 0.080 | 27.211 |  |  |  |
| T19 | 31.415 | 27.175 |  |  |  |  |  |
| T20 | 31.389 | 27.399 | 0.070 | 31.423 | 3.923 | 0.331 | 1.258 |
| T20 | 31.503 | 27.660 | 0.140 | 27.500 |  |  |  |
| T20 | 31.377 | 27.442 |  |  |  |  |  |
| T21 | 27.245 | 24.376 | 0.750 | 27.944 | 3.535 | 0.719 | 1.646 |
| T21 | 27.850 | 24.388 | 0.046 | 24.408 |  |  |  |
| T21 | 28.736 | 24.461 |  |  |  |  |  |
| T22 | 29.605 | 26.654 | 1.866 | 29.144 | 1.968 | 2.285 | 4.875 |
| T22 | 27.091 | 27.571 | 0.471 | 27.176 |  |  |  |
| T22 | 30.737 | 27.302 |  |  |  |  |  |
| N1 | 33.716 | 29.603 | 0.817 | 33.252 | 3.968 | 0.286 | 1.219 |
| N1 | 33.731 | 29.634 | 0.579 | 29.284 |  |  |  |
| N1 | 32.309 | 28.616 |  |  |  |  |  |
| N2 | 28.389 | 25.812 | 3.549 | 30.647 | 4.829 | -0.575 | 0.671 |
| N2 | 28.815 | 25.673 | 0.149 | 25.819 |  |  |  |
| N2 | 34.738 | 25.971 |  |  |  |  |  |
| N3 | 29.884 | 26.525 | 0.332 | 30.079 | 3.506 | 0.748 | 1.680 |
| N3 | 29.890 | 26.314 | 0.287 | 26.573 |  |  |  |
| N3 | 30.462 | 26.881 |  |  |  |  |  |
| N4 | 30.933 | 26.094 | 1.435 | 29.647 | 3.798 | 0.456 | 1.372 |
| N4 | 28.099 | 25.903 | 0.276 | 25.849 |  |  |  |
| N4 | 29.908 | 25.550 |  |  |  |  |  |
| N5 | 34.976 | 30.168 | 1.015 | 34.127 | 3.500 | 0.754 | 1.686 |
| N5 | 34.401 | 31.171 | 0.507 | 30.627 |  |  |  |
| N5 | 33.003 | 30.542 |  |  |  |  |  |
| N6 | 33.798 | 30.997 | 0.283 | 33.561 | 3.289 | 0.965 | 1.952 |
| N6 | 33.639 | 29.548 | 0.725 | 30.272 |  |  |  |
| N6 | 33.248 | 30.272 |  |  |  |  |  |
| N7 | 31.942 | 28.930 | 3.184 | 33.666 | 4.472 | -0.219 | 0.859 |
| N7 | 31.717 | 29.089 | 0.329 | 29.194 |  |  |  |
| N7 | 37.341 | 29.563 |  |  |  |  |  |
| N8 | 32.760 | 26.830 | 3.075 | 29.551 | 2.920 | 1.334 | 2.520 |
| N8 | 29.263 | 26.247 | 0.332 | 26.631 |  |  |  |
| N8 | 26.630 | 26.815 |  |  |  |  |  |
| N9 | 32.700 | 28.331 | 0.542 | 32.800 | 4.469 | -0.216 | 0.861 |
| N9 | 32.315 | 28.444 | 0.113 | 28.331 |  |  |  |
| N9 | 33.386 | 28.219 |  |  |  |  |  |
| N10 | 30.635 | 27.314 | 1.795 | 31.828 | 4.806 | -0.552 | 0.682 |
| N10 | 30.956 | 26.972 | 0.271 | 27.022 |  |  |  |
| N10 | 33.892 | 26.779 |  |  |  |  |  |
| N11 | 30.265 | 27.946 | 2.381 | 32.646 | 5.174 | -0.920 | 0.529 |
| N11 | 32.646 | 27.079 | 0.439 | 27.472 |  |  |  |
| N11 | 35.028 | 27.392 |  |  |  |  |  |
| N12 | 34.291 | 28.675 | 1.319 | 32.797 | 4.086 | 0.168 | 1.123 |
| N12 | 32.306 | 28.756 | 0.042 | 28.711 |  |  |  |
| N12 | 31.794 | 28.701 |  |  |  |  |  |
| N13 | 33.498 | 25.968 | 0.594 | 32.871 | 6.928 | -2.674 | 0.157 |
| N13 | 32.316 | 25.841 | 0.093 | 25.943 |  |  |  |
| N13 | 32.800 | 26.021 |  |  |  |  |  |
| N14 | 31.766 | 26.991 | 1.583 | 31.887 | 4.798 | -0.545 | 0.686 |
| N14 | 33.527 | 26.846 | 0.303 | 27.089 |  |  |  |
| N14 | 30.368 | 27.428 |  |  |  |  |  |
| N15 | 27.486 | 28.638 | 4.181 | 31.994 | 3.269 | 0.985 | 1.979 |
| N15 | 32.749 | 28.811 | 0.087 | 28.724 |  |  |  |
| N15 | 35.746 | 28.724 |  |  |  |  |  |
| N16 | 31.971 | 28.482 | 2.286 | 32.860 | 4.249 | 0.005 | 1.003 |
| N16 | 31.153 | 29.053 | 0.394 | 28.611 |  |  |  |
| N16 | 35.457 | 28.297 |  |  |  |  |  |

Table S7 16 overlapping mRNAs between module genes and CNC network genes

| mRNA | Fold Change | Regulation | P value |
| --- | --- | --- | --- |
| CHST6 | 2.3253412 | down | 0.007301865 |
| USP9Y | 2.6109927 | down | 0.02366001 |
| PPP1R12A | 4.1554052 | down | 0.003294782 |
| IDS | 2.8012837 | down | 0.039357423 |
| GPC3 | 2.437132 | down | 0.024478276 |
| PLCB4 | 2.2909807 | down | 0.024902135 |
| KATNB1 | 2.1231421 | down | 0.031600868 |
| MYL3 | 2.2624683 | down | 0.021622199 |
| RBM20 | 2.2102025 | down | 0.03517652 |
| ABHD5 | 2.1801143 | down | 0.011005913 |
| NDUFV1 | 3.1494792 | down | 0.001312557 |
| TCTN1 | 2.1331862 | up | 0.018517392 |
| PRR5 | 2.9576901 | up | 0.004192674 |
| CEP85 | 2.1979472 | up | 0.005519906 |
| SAYSD1 | 2.1091626 | up | 0.002939776 |
| NR2F2 | 2.0174502 | up | 0.001459818 |

Figure S1 The receiver operating characteristic (ROC) curves of the combination model of three lncRNAs


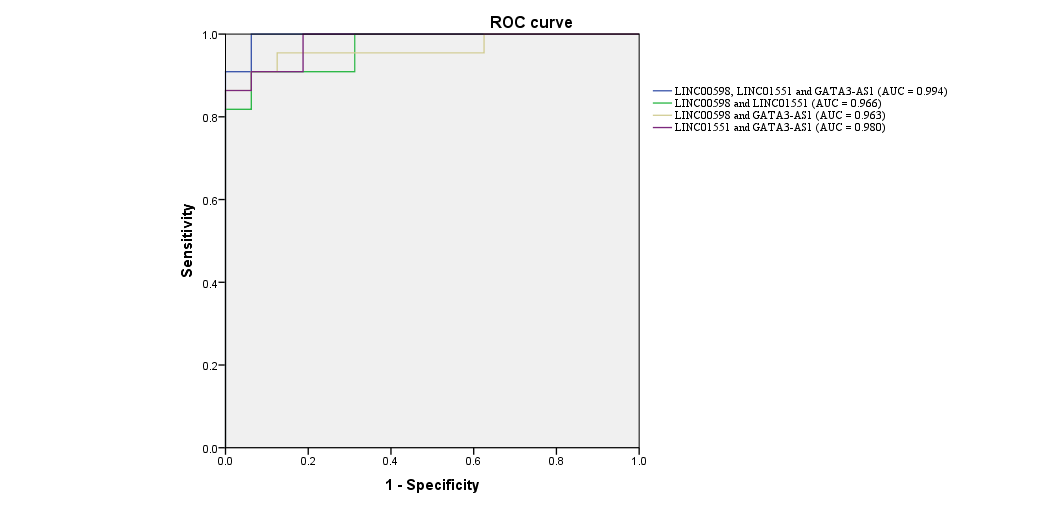


ROC: Receiver operating characteristic; AUC: Area under the ROC curve
